# The Fungus among Us: *Cryptococcus neoformans* and *Cryptococcus gattii* Ecological Modeling for Colombia

**DOI:** 10.3390/jof1030332

**Published:** 2015-09-30

**Authors:** Sunny Mak, Nórida Vélez, Elizabeth Castañeda, Patricia Escandón, Colombian Environmental Study Group

**Affiliations:** 1 Public Health Analytics, British Columbia Centre for Disease Control, 655 West 12th Avenue, Vancouver, BC V5Z 4R4, Canada; 2 Grupo de Microbiología, Instituto Nacional de Salud, Av. Calle 26 No. 51-20, Bogotá, D.C. 111321, Colombia; E-Mails: noridavelezc@gmail.com (N.V.); ecastaneda21@gmail.com (E.C.); pescandon@ins.gov.co (P.E.); 3 Catalina de Bedout, Luz Elena Cano, Corporación para Investigaciones Biológicas, Medellín, Antioquia 055428, Colombia; 4 María Clara Noguera, Universidad Metropolitana, Barranquilla, Atlántico 080001, Colombia; 5 Fabiola González, Cristian Anacona, Universidad del Cauca, Popayán, Cauca 111321, Colombia; 6 Jairo Lizarazo, Hospital Universitario Erasmo Meoz, Cúcuta, Norte de Santander 540004, Colombia; 7 María Inés Alvárez, Universidad del Valle, Cali, Valle del Cauca 025360, Colombia

**Keywords:** Cryptococcus, cryptococcal disease, ecological niche modeling, risk mapping

## Abstract

The environmental isolation of *Cryptococcus* spp. is typically a difficult undertaking. Collecting samples in the field is costly in terms of travel, personnel time and materials. Furthermore, the recovery rate of *Cryptococcus* spp. may be very low, thereby requiring a large number of samples to be taken without any guarantee of success. Ecological niche modeling is a tool that has traditionally been used to forecast the distribution of plant and animal of species for biodiversity and conservation purposes. Here, we use it in a public health application to produce risk area maps for cryptococcal disease in Colombia. The Genetic Algorithm for Ruleset Production (GARP) was used to create models for *Cryptococcus neoformans* (*C. neoformans*) and *Cryptococcus gattii* (*C. gattii*), based on environmental sampling and clinical records data recorded since 1987. These maps could be used to focus public health messaging related to cryptococcal disease, and it enables us to characterize the ecological niche for *Cryptococcus* in Colombia. We found that the ecological niche for *C. gattii* in Colombia is quite diverse, establishing itself in sub-tropical and temperate ecoregions within the country. This suggests that *C. gattii* is highly adaptive to different ecological conditions in Colombia and different regions of the world.

## 1. Introduction

*Cryptococcus* is a fungal organism (yeast cells or blastoconidia ~1–2 micrometers in size) that can cause illness in humans and animals through environmental exposure. Of particular medical interest are *Cryptococcus neoformans* (serotypes A, AD and D) and *Cryptococcus gattii* (serotypes B and C). *Cryptococcus neoformans* (*C. neoformans*) has a worldwide distribution and is typically found in bird droppings in cosmopolitan settings; those infected by *C. neoformans* are predominantly immunocompromised [1]. Conversely, the geographical distribution of *Cryptococcus gattii* (*C. gattii*) was traditionally understood to be limited to tropic and sub-tropic regions in association with *Eucalyptus* spp. trees [2]; however, emergence of *C. gattii* in the Pacific Northwest region of North America, a temperate rainforest region, has redefined the ecological niche of *C. gattii* [3,4]. The majority of *C. gattii* infections occur in immunocompetent persons.

Risk maps are useful for delineating the geographical areas where potential exposure to environmental pathogens may occur. The purpose of this study was to create ecologically-based risk maps for *C. neoformans* and *C. gattii* for Colombia to assist clinicians in the diagnosis of cryptococcal disease, and to direct public health messaging related to cryptococcal disease. This study also describes the environmental characterization of the ecological niche for *C. neoformans* and *C. gattii* for Colombia based on the environmental predictor data used in the modeling.

Previous clinical and epidemiological research performed by the Colombian Group for the Study of Cryptococcosis identified an increased incidence related to the AIDS pandemic. The mean annual incidence of cryptococcal disease in the general population between 1997–2005 and 2006–2010 remained the same at 2.4 cases per million population; whereas, in AIDS patients the incidence rate rose from 3000 cases per million population during 1997–2005 up to 3300 cases per million population during 2006–2010. The vast majority of clinical isolates recovered (*n* = 1074; 96.6%) belong to *C. neoformans* var. *grubii* (serotype A), five isolates (0.4%) to *C. neoformans* var. *neoformans* (serotype D), and 33 isolates (3.0%) to *C. gattii*, with Norte de Santander the department with the highest frequency of cryptococcosis cases (77%) caused by *C. gattii* in the immunocompetent population [5,6].

Several environmental approaches have been undertaken in Colombia to describe the environmental importance of the fungus and its potential association with clinical isolates. These reports have documented the isolation of *C. neoformans* var. *grubii* and *C. gattii* from diverse sources, such as *Eucalyptus* spp., *Ficus* spp*.* and *Terminalia catappa* trees, among others [7,8,9]. Analysis of environmental climatic factors has identified higher frequency and density of *C. neoformans* in the rainy season (particularly during the wet and humid months of April and May) than in the dry season [10], but a differential relationship between *C. gattii* serotypes B and C and humidity, temperature, evaporation and solar radiation in Colombia was also described [11].

Risk maps are typically the outcomes of models of disease transmission based on epidemiological, climatic and environmental data [12]. These models can be informed by expert opinion based on coarse scale datasets, created using statistical algorithms and geographic information systems (GIS) modeling based on spatially refined datasets, or based on observations from field sampling. The development of a cryptococcal disease risk map is challenging because the environmental isolation of *Cryptococcus* spp. is a difficult undertaking. Collecting samples in the field, and subsequent testing in the laboratory, is costly in terms of travel, personnel time and materials; *C. neoformans* and *C. gattii* cannot be identified with the naked eye, and these fungal organisms are not believed to adversely affect the health of its plant hosts. Furthermore, the recovery rate of *Cryptococcus* spp. may be very low, thereby requiring a large number of samples to be taken without any guarantee of success [13].

However, when the information is available, disease risk maps generated from ecological niche modeling can provide accurate forecasts of a pathogenic organism’s ecological niche based on the environmental characteristics of its observed locations [14]. Supporting this work is the increased availability and quality of environmental data derived from remote sensing (*i.e.*, aerial or satellite imagery) sources, and compatibility with GIS and species distribution modeling software [12]. Environmental predictor layers used in ecological niche modeling comprise of biotic (vegetation, animal, microorganism) and/or abiotic (climate, terrain, soil) data.

The concept of the ecological niche of a species is defined as the set of physical and biological conditions under which the species can maintain its population without immigration [15]. For a species to maintain its population, individuals must be able to survive and reproduce. The fundamental niche of a species consists of the total potential area that meets all the physical and biological requirements of a species (*i.e.*, geographical space and environmental components); whereas, the realized niche of a species is comprised of the actual distribution of a species determined by a variety of factors such as dispersal, history, and physical barriers (*i.e.*, geographical space, environmental components and species responses) [16,17,18]. Ecological niche modeling therefore uses observations of a species’ occurrences from its realized niche, and produces a forecast of the species’ fundamental niche.

The use of clinical records for disease risk mapping and assessment has proved useful in some applications [14,19] but it also introduces model uncertainty because the place of environmental exposure to the infectious agent may be different than the case’s location of residence (*i.e.*, potential misclassification of the place of species occurrence). Ideally, the disease risk model would be based on environmental findings collected under a random location-sampling scheme with a high number of species presence observations from a wide geographical area. Environmental sampling results are heavily dependent on sampling effort though. Repeat sampling is often performed at historic presence locations or near the environs of reported cases. This produces reassuring results for the investigators, but from an ecological niche modeling/risk map creation perspective, it does not produce additional data points to inform the creation of the model. The likelihood of obtaining positive *Cryptococcus* spp. results from random sampling however are typically very low [20]. Furthermore, in the context of an emerging pathogen or geographical range expansion, the investigator must determine whether a single positive environmental observation is sufficient to determine the established presence of *Cryptococcus* spp. or if replicate findings after a period of time is necessary. Additionally, it is difficult to determine how many negative environmental sampling results are sufficient to classify an area as having a definitive absence of an organism.

## 2. Experimental Section

Environmental and clinical data were managed in an Excel table (Microsoft Corp, Redmond, WA, USA) and imported into a GIS (ArcGIS 10.0, Redlands, CA, USA) for mapping and analysis. Approximate latitude and longitude coordinates from where the environmental samples were collected were derived from Google Maps (Google, Mountain View, CA, USA). Environmental sampling data were gathered from the information collected by the Colombian Microbiology Group of the National Institute of Health for the period 1987–2011; additionally, extensive environmental sampling was performed in 13 geographical departments of Colombia during the years 2012–2014 for the purpose of increasing the number of input data points to support the ecological niche modeling (Supplementary Table S1). Environmental sampling consisted of collecting plant material from different species of trees and biological samples from bird excreta. Sampling was conducted in cities where cryptococcosis cases had been reported, specifically in parks and urban areas with high density of native and introduced species of trees, as well as places where the presence of bird excreta was observed. Samples were processed using conventional techniques, inoculating the supernatant on *Guizottia abyssinica* agar plates supplemented with Byphenil and antibiotics. Plates were observed daily during one month, and suspected brown colonies were streaked onto Sabouraud glucose agar plates for further phenotypic characterization and species determination [21]. Additionally, one veterinary case was included, from a *Canis lupus familiaris.* Clinical cases recorded in the National Epidemiology Survey (a passive disease surveillance system) were geocoded to their address of residence.

Numerous ecological niche-modeling algorithms are available (envelope models, maximum entropy, rule-based, splines, classical statistics) [22]. However, fundamentally, ecological niche models are correlative models—they relate observed presences of a species to values of environmental variables at those location. We used Desktop GARP [23] because of its utility in a wide range of applications, strong user support, ability to produce reliable models with a small number of input data points and consistency with previous analyses [14,24]. Detailed description of the GARP methodology is available elsewhere [25]. In summary, GARP uses a “superset” of rules to identify the ecological niche of a species based on non-random correlations among species presence, species absence and environmental parameter values [23]. The species occurrence data are split into training and testing subsets, and the environmental data layers believed relevant to the ecology of the species in question are used to construct the model. The rules for predicting the ecological niche of a species—atomic, range, negated rate and logistic regression—are randomly developed and progressively applied on the training dataset. The rule is accepted and incorporated into the model if the change in predictive accuracy increases; otherwise, the rule is rejected and dropped [24].

Predictive accuracy of the model is calculated by dividing the number of correct predictions (*i.e.*, predicted presence—observed presence and predicted absence—observed absence) by the total number of predictions (*i.e.*, sum of correct and incorrect predictions). A commission error occurs when the model predicts a species to occur where it does not, and an omission error occurs when the model fails to predict a species occurrence where it does in fact occur.

**Figure 1:**
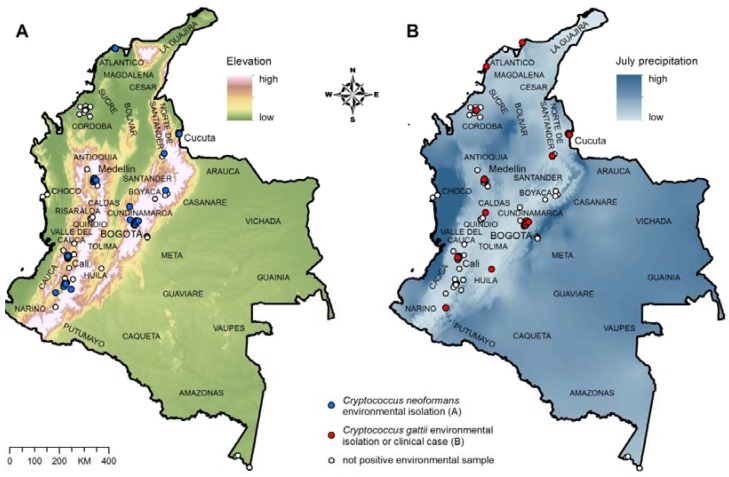
(**A**) Environmental isolation of *C. neoformans*; (**B**) Environmental isolation and clinical cases of *C. gattii* infection. The departments of Colombia are labeled on the map.

We used 60 records of *C. neoformans* environmental isolations (Figure 1A) to build and test the ecological niche model for *C. neoformans*, and 36 records of *C. gattii* environmental isolations (11 records) and clinical cases (25 records; to increase the number of data points for the modeling; Figure 1B) to build and test the ecological niche model for *C. gattii*.

Further validation of the C. neoformans model was accomplished by overlaying the clinical reports of cryptococcal disease due to C. neoformans (51 records) to verify the predictive accuracy of the model. Details of the isolations of *C. neoformans* and *C. gattii* used to create the ecological niche models are described in Supplementary Table S2.

We used 38 environmental predictor layers (Table 1) believed to be relevant to *C. neoformans* and *C. gattii* biogeography in the ecological niche modeling: temperature, precipitation and elevation layers from WorldClim [26], land cover from the European Environment Agency [27] and the Food and Agricultural Organization of the United Nations (FAO) [28], soil type from the FAO [29] and maximum green vegetation fraction (2001–2012 average) from the United States Geological Survey [30]. The spatial resolution of these datasets is 1 km with geographical coverage over the entire extent of Colombia. Slope and aspect direction were calculated from the elevation data layer using the Spatial Analyst extension of ArcGIS. A jack-knifing procedure was performed to identify environmental layers that are good predictors for *Cryptococcus* spp. using Desktop GARP. The *C. neoformans* and *C. gattii* input datasets were split into 50% training and 50% testing subsets to build and test the models. The environmental data layers were jack-knifed individually through 30 model runs for *C. neoformans* and *C. gattii*. Each model run comprised of up to 1000 iterations or until convergence was reached.

**Table 1:** List of environmental data layers used in the ecological niche models. Data layers with ≥80% training and testing accuracy for *C. neoformans* and *C. gattii* from the jack-knifing procedure are indicated (“yes”).

Layer	Source	*C. neoformans* Model	*C. gattii* Model
Annual mean temperature	WorldClim	-	-
Mean diurnal range		-	-
Isothermality		-	-
Temperature seasonality		-	-
Maximum temperature of warmest month		-	-
Minimum temperature of coldest month		-	-
Temperature annual range		-	-
Mean temperature of wettest quarter		-	-
Mean temperature of driest quarter		-	-
Mean temperature of warmest quarter		-	-
Mean temperature of coldest quarter		-	-
Annual precipitation		-	yes
Precipitation of wettest month		-	yes
Precipitation of driest month		-	-
Precipitation seasonality		-	-
Precipitation of wettest quarter		-	yes
Precipitation of driest quarter		-	-
Precipitation of warmest quarter		-	-
Precipitation of coldest quarter		-	yes
January precipitation		-	-
April precipitation		yes	yes
July precipitation		yes	yes
October precipitation		-	-
Mean January maximum temperature		-	-
Mean April maximum temperature		-	-
Mean July maximum temperature		-	-
Mean October maximum temperature		-	-
Mean January minimum temperature		-	-
Mean April minimum temperature		-	-
Mean July minimum temperature		yes	-
Mean October minimum temperature		yes	-
Elevation		yes	-
Aspect	derived from WorldClim	-	-
Slope		-	-
Global land cover—2000	Europa	-	-
Global land cover—SHARE 2014	FAO	-	yes
Dominant soil type		-	-
Maximum green vegetation fraction	USGS	-	yes

Environmental data layers with training and testing accuracies ≥80% were used to create the final ecological niche models for *C. neoformans* and *C. gattii*. For these final model runs, the input *C. neoformans* and *C. gattii* data were again divided into training (50%) and testing (50%) subsets, and 50 model runs were performed. Each model run produced a binary value output data layer with 1 indicating areas with suitable ecological conditions to support *C. neoformans* and *C. gattii*, respectively, and 0 indicating unsuitable areas. The 50 output data layers where overlaid and summed to produce a final, composite ecological niche map for *C. neoformans* and *C. gattii*.

Lastly, we used our expert opinion to critically assess the predicted ecological niche model output for *C. neoformans* and *C. gattii*. We reclassified geographical areas above 3000 m elevation as “not suitable” for *C. neoformans* and *C. gattii* because the Páramo climate altitudinal zone (3000–4000 m above sea level) is characterized by temperature below 10 °C with icy winds and frequent snowfall, and the glacial climate altitudinal zone (>4000 m) is characterized by glaciers [31]. These conditions are not believed to be conducive for *C. neoformans* and *C. gattii* based on previous environmental sampling findings in Colombia, and observations from Vancouver Island, Canada [11,14,32].

## 3. Results and Discussion

**Figure 2:**
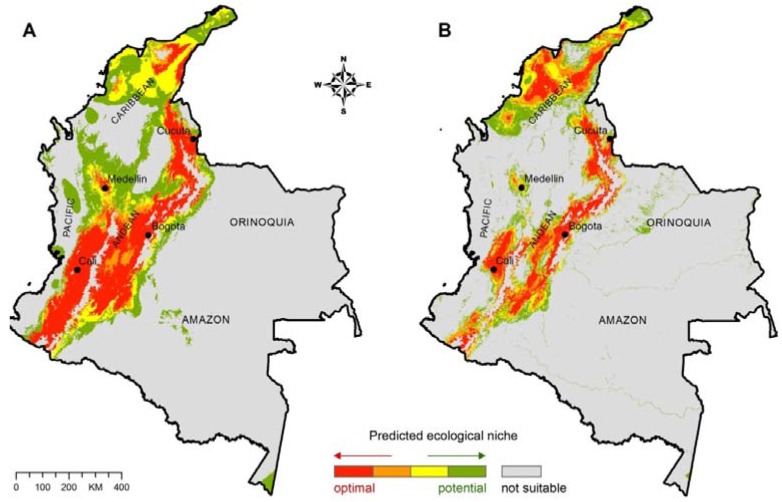
(**A**) Ecological niche model for *C. neoformans*; (**B**) Ecological niche model for *C. gattii.* The major ecoregions of Colombia are labeled on the map.

April and July precipitation, mean July and October minimum temperature, and elevation had training and testing accuracy ≥80% based on the jack-knifing procedure of environmental data layers against the distribution of *C. neoformans* environmental isolations (Table 1; Figure 1A). Annual, April and July precipitation, precipitation of the wettest month, wettest quarter and coldest quarter, land cover, and maximum green vegetation fraction had training and testing accuracy ≥80% based on the jack-knifing procedure of environmental data layers against the distribution of *C. gattii* environmental isolations and clinical cases (Table 1; Figure 1B). These environmental data layers were used to produce the final ecological niche models for *C. neoformans* (Figure 2A) and *C. gattii* (Figure 2B).

The prediction model for *C. neoformans* in Colombia revealed the presence of optimal ecological niche areas along the mountainous Andean natural region, extending from the department of Cauca in western Colombia through to the department of Magdalena in northwestern Colombia (Figure 2A). This mountain range is comprised of several heavily populated departments such as Cundinamarca (Bogotá, capital city), Boyacá and Santander, and an important number of cryptococcosis cases have been reported from this region [6]. Furthermore, numerous environmental isolations of *C. neoformans* have been detected in this region (Figure 1A). Potentially, the ecological niche model suggests that the fungus may be present in very low, not yet detected concentration, or may disperse in the future to the Caribbean region along the northern coast of the country. The prediction model for *C. neoformans* in Colombia had a final training accuracy of 91.6% and testing accuracy of 86.4%.

The predicted ecological niche areas for *C. gattii* follow a similar, but less extensive, geographical distribution (Figure 2B). These regions are generally characterized as the warm climate altitudinal zone positioned between sea level and 1000 m elevation, having temperatures above 24 °C and heavy rains (the cities of Cali and Cúcuta for example), the temperate climate altitudinal zone positioned between 1000 and 2000 m above sea level, having temperatures between 17 and 22 °C, and annual precipitation between 2000 and 2500 mm (the city of Medellin for example), and the cold climate altitudinal zone positioned between 2000 and 3000 m above sea level, having temperatures between 10 and 17 °C, and annual precipitation above 2000 mm (the city of Bogotá for example) [33]. The prediction model for *C. gattii* in Colombia had a final training accuracy of 92.9% and testing accuracy of 89.1%.

Not suitable ecological niche areas for *C. gattii* and *C. neoformans* include the tropical rainforest regions (hot and high humidity climate with heavy rainfall) along the Pacific coast and Amazon River basin, and the Orinoquia region in western Colombia (Figure 2A,B).

Overall, the environmental characterization of *C. neoformans* and *C. gattii* based on empirical data correlated with the environmental characterization of *C. neoformans* and *C. gattii* based on prediction modeling (Table 2). The minor to moderate variations between empirical data and prediction modeling reflect the iterative, evolving nature of the decision rule based GARP algorithm. Precipitation (April, July, annual, wettest month, wettest quarter, coldest quarter) appears to have a major role in determining the ecological niche for *C. gattii*.

The environmental data layers used for the creation of the model show that Colombia has a group of macro-ecologic variables where the presence of the fungi is suitable. The country of Colombia is strategically positioned in the South American continent, crossed by the Equatorial zone, which determines the existence of a wide variety of climates and ecosystems; therefore, the country does not have traditional seasons, but instead only a rainy winter and a dry summer.

**Table 2:** Environmental characterization of *C. neoformans* based on field observations and *C. gattii* based on field observations and clinical reports, and environmental characterization of *C. neoformans* and *C. gattii* based on ecological niche modeling (top quartile of models).

*C. neoformans*	Based on Field Observations			Based on Ecological Niche Modeling		
Layer	Minimum	Mean	Maximum	Minimum	Mean	Maximum
Elevation (m)	9	1425	2618	291	1673	3542
April precipitation (mm)	68	141	360	25	156	455
July precipitation (mm)	24	75	471	4	64	151
Mean July minimum temperature (°C)	8.0	15.4	22.9	3.3	13.6	22.9
Mean October minimum temperature (°C)	8.3	15.3	22.8	3.5	13.9	22.5
***C. gattii***	**Based on Field Observations and Clinical Reports**			**Based on Ecological Niche Modeling**		
Layer	Minimum	Mean	Maximum	Minimum	Mean	Maximum
April precipitation (mm)	17	117	224	4	117	338
July precipitation (mm)	24	57	154	1	75	223
Annual precipitation (mm)	529	1016	1951	437	1095	1868
Precipitation of wettest month (mm)	101	147	267	59	159	357
Precipitation of wettest quarter (mm)	246	365	706	166	411	813
Precipitation of coldest quarter (mm)	31	234	634	1	226	702
Maximum green vegetation fraction (%)	23	58	99	1	90	100
Land cover	82% grassland, 12% tree cover, 3% cropland, 3% sparse vegetation			48% tree cover, 34% grassland, 14% cropland, 2% shrubs, 1% waterbodies, 1% other		

A limitation of this study is the paucity of environmental sampling data from much of the rural and remote areas of Colombia such as the vast Amazonas and Orinoquia regions. Extensive sampling (>18000 environmental samples) was performed in 13 departments where cases of cryptococcal disease have been reported, where *C. neoformans* and *C. gattii* have been previously isolated in the environment, and in areas of particular epidemiological and ecological interest (Figure 1; Supplementary Table S1). Environmental sampling was performed in a small area of the Amazonas, which yielded no positive findings; unfortunately, it was not feasible to conduct more extensive environmental sampling in the other regions of the Amazonas or in the Orinoquia region for this study. We acknowledge that absence of data from these regions have the potential to negatively affect the prediction model because data in the pluvial forest regions excludes these environmental parameters from the final model; however, the predictions presented in this study are the best available based on the empirical data collected over the past 10 years.

Another limitation of this study is the use of *C. gattii* clinical case data to augment the environmental observations for the predictive modeling. This introduces uncertainty as to whether the case’s place of residence was in fact the place of exposure to *C. gattii* in the environment, and it has the potential to result in modeling error. However, Mak *et al.* [14] have demonstrated the successful use of ecological niche modeling based on human and animal *C. gattii* disease surveillance data. Furthermore, validation of the *C. neoformans* model based on field observations was accomplished by overlaying the clinical reports of cryptococcal disease due to *C. neoformans* (The *C. neoformans* clinical dataset was not used to train or test the *C. neoformans* prediction model based on field observations). Of the 51 clinical records, 50 were located within the forecasted ecological niche of *C. neoformans*, thereby further validating the predictive accuracy of the model.

Further refinement of the prediction models may include dividing *C. neoformans* into *C. neoformans* var. *grubii* and *C. neoformans* var. *neoformans* as separate species, and the five species within *C. gattii*—in recognition of the new taxonomy of the *C. neoformans*/*C. gattii* species complex recently proposed by Hagen and colleagues [34]. However, this may only be possible if sufficient data are available to train and test the new models. Additional sampling, especially from geographically diverse locations throughout Colombia, would be required.

Lastly, it is important to note that the prediction models for *C. neoformans* and *C. gattii* describe the ecological niche areas for these fungal species at the regional scale. The actual habitat suitability and ultimate presence of *C. neoformans* and *C. gattii* at a specific location at the site level is highly variable because there will most likely be considerable ecological heterogeneity within each 1 km by 1 km spatial unit in the ecological niche model. Instead, the maps can be used by clinicians to consider the diagnosis of cryptococcal disease for patients residing in or having recent travel to these geographical areas.

## 4. Conclusions

In this study we describe the utility of ecological niche modeling to produce risk maps for *C. neoformans* and *C. gattii* for Colombia. We found that the ecological niche for *C. gattii* in Colombia is quite diverse, establishing itself in sub-tropical and temperate ecoregions within the country. This suggests that *C. gattii* is highly adaptive to different ecological conditions in Colombia and different regions of the world. Furthermore, the modeling software (Desktop GARP) and environmental predictor layers used in this study are available at no cost, and the methodology is applicable to other jurisdictions and fungal organisms.

## Supplementary Materials

Supplementary File 1
