# Supplementary material for: The Fungus among Us: Cryptococcus neoformans and Cryptococcus gattii Ecological Modeling for Colombia
Source: J Fungi (Basel). 2015 Sep 30;1(3):332–44. doi: 10.3390/jof1030332 (PMC5753128; doi:10.3390/jof1030332)
Supplement: Supplementary File 1 [file jof-01-00332-s001.pdf]

Supplementary Materials

Table S1. Summary of environmental sampling for *C. neoformans* and *C. gattii* by department.

Department	Positive n (%)	Not Positive n (%)	Total
Antioquia	19 (0.6)	2879 (99.4)	2898
Amazonas	0 (0.0)	310 (100.0)	310
Atlántico	9 (0.5)	1935 (99.5)	1944
Bogotá	35 (0.8)	4081 (99.2)	4116
Boyacá	1 (0.1)	884 (99.9)	885
Cauca	308 (12.5)	2159 (87.5)	2467
Chocó	0 (0.0)	300 (100.0)	300
Córdoba	0 (0.0)	782 (100.0)	782
Nariño	1 (0.1)	688 (99.9)	689
Norte de Santander	12 (0.6)	1891 (99.4)	1903
Meta	0 (0.0)	300 (100.0)	300
Risaralda	0 (0.0)	368 (100.0)	368
Valle	16 (1.0)	1579 (99.0)	1595
Total	401 (2.2)	18,156 (97.8)	18,557

Table S2. *C. neoformans* and *C. gattii* clinical and environmental isolates used for the creation of the ecological niche models.

Isolate H0058-I-	Year Isolation	Geographical Origin	Source
	***C. neoformans* clinical isolates**		
894	1997	Bogotá DC	CSF
939	1998	Santander	CSF
967		Norte de Santander	CSF
995	1999	Norte de Santander	CSF
1091		Santander	CSF
997		Risaralda	CSF
1193	2000	Norte de Santander	CSF
1226			CSF
1328		Huila	CSF
No viable strain		Norte de Santander	CSF
1398	2001	Bogotá DC	Lung biopsy
1394			CSF
1256			CSF
1508	2002	Bogotá DC	CSF
1510		Norte de Santander	CSF
1548		Bogotá DC	CSF
1917	2003	Cundinamarca	CSF
1918		Cundinamarca	CSF
1646		Bogotá DC	CSF
2206	2004	Huila	CSF
2734	2006	Bogotá DC	CSF
2583		Antioquia	CSF
2843			CSF
2836		Nariño	CSF
2681		Bogotá DC	CSF
2933	2007	Bogotá DC	Blood
2955			CSF
4640			CSF
3089		Antioquia	CSF
2778			CSF
2792		Norte de Santander	CSF
3099	2008	Valle	CSF
3199	2009	Bogotá DC	CSF
3196			CSF
3224			Blood
3182		Antioquia	CSF
3212	2010	Antioquia	CSF
5031		Norte de Santander	CSF
3744	2011	Antioquia	CSF

Table 2. *Cont.*

Isolate H0058-I-	Year Isolation	Geographical Origin	Source
3848	2012	Risaralda	CSF
3820		Antioquia	CSF
4378	2013	Valle	CSF
3820		Antioquia	CSF
3952	2014	Norte de Santander	Body Fluid
5790		Antioquia	CSF
5382		Norte de Santander	CSF
5700			CSF
5684			CSF
5913			CSF
5919			CSF
5127			CSF
C. neoformans **environmental isolates**			
No viable strain *	1987	Valle	*Columba livia*
No viable strain		Bogotá DC	*Columba livia*
No viable strain			*Columba livia*
No viable strain			*Columba livia*
No viable strain	1989	Cundinamarca	*Columba livia*
991	1999	Bogotá DC	*Columba livia*
1247	2001	Bogotá DC	*Eucalyptus* spp.
1602	2002	Norte de Santander	*Columba livia*
1973	2003	Antioquia	*Eucalyptus* spp.
1660		Bogotá DC	*Columba livia*
No viable strain	2004	Antioquia	*Eucalyptus* spp.
No viable strain			*Eucalyptus* spp.
2636	2006	Bogotá DC	*Eucalyptus* spp.
2614		Santander	*Terminalia catappa*
2872	2007	Bogotá DC	*Corymbia ficifolia*
3082	2008	Norte de Santander	*Terminalia catappa*
3692	2011	Antioquia	*Acacia* spp.
3593		Bogotá DC	*Corymbia ficifolia*
3527		Cundinamarca	*Eucalyptus* spp.
3671		Bogotá DC	*Eucalyptus* spp.
3554			*Eucalyptus* spp.
3656		Norte de Santander	*Terminalia catappa*

Table 2. *Cont.*

Isolate H0058-I-	Year Isolation	Geographical Origin	Source
3973	2012	Cauca	*Columba livia*
4013			*Tabebuia guayacan*
3900			*Acacia* spp.
4050		Antioquia	*Terminalia catappa*
4052			*Hibiscus*
3907			*Eucalyptus* spp.
3906		Norte de Santander	*Terminalia catappa*
4272	2013	Cauca	*Columba livia*
4600			*Columba livia*
4517			*Columba livia*
4253			*Columba livia*
4517			*Columba livia*
4474			*Columba livia*
4080			*Eucalyptus* spp.
4092			*Columba livia*
4462			*Columba livia*
4141			*Columba livia*
4314			*Columba livia*
4291			*Eggretta thula*
4555			*Columba livia*
4573			*Columba livia*
4393		Valle	*Columba livia*
4417			*Samanea saman*
4872			*Roystonea regia*
4606		Antioquia	*Acacia* spp.
4826			*Terminalia catappa*
4706		Atlántico	*Terminalia cattapa*
4712			*Terminalia cattapa*
4697			*Pink trumpet*
4455		Bogotá DC	*Eucalyptus* spp.
4372			*Eucalyptus* spp.
4872		Valle	*Caesalpinia*
5376	2014	Cauca	*Columba livia*
5353		Antioquia	*Terminalia catappa*
5409		Bogotá DC	*Eucalyptus* spp.
5411			*Eucalyptus* spp.
6073		Boyacá	*Eucalyptus* spp.
5545		Norte de Santander	*Terminalia catappa*

Table 2. *Cont.*

Isolate H0058-I-	Year Isolation	Geographical Origin	Source
**C. gattii clinical isolates**			
107	1990	Norte de Santander	CSF
239	1993	Norte de Santander	CSF
255	1994	Norte de Santander	CSF
1278	2001	Norte de Santander	CSF
1708	2003	Bogotá DC	CSF
2746	2007	Valle	CSF
2792		Norte de Santander	CSF
2802		Antioquia	Pharynx
2877		Norte de Santander	CSF
2858		Norte de Santander	CSF
3031	2008	Bogotá DC	CSF
3032		Bogotá DC	CSF
3043		Norte de Santander	CSF
3096		Antioquia	CSF
3146	2009	Norte de Santander	CSF
3407		Norte de Santander	CSF
3172		Valle	CSF
3266	2010	Norte de Santander	CSF
3286		Antioquia	BAL
3407		Norte de Santander	CSF
3590	2011	Antioquia	Blood
3826	2012	Atlántico	Blood
5126	2014	Valle	CSF
5670	2014	Cauca	CSF
6082	2014	Cauca	CSF
**C. gattii environmental isolates**			
1942	2003	Antioquia	*Eucalyptus* spp.
2202-1	2004	Antioquia	*Eucalyptus* spp.
2445	2005	Bogotá DC	*Eucalyptus* spp.
2751	2006	Bogotá DC	*Eucalyptus* spp.
2872	2007	Bogotá DC	*Corymbia ficifolia*
3327	2010	Norte de Santander	*Terminalia catappa*
3298	2010	Norte de Santander	*Terminalia catappa*
3504	2011	Huila	*Terminalia catappa*
3684	2011	Bogotá DC	*Rose apple*
3523	2011	Bogotá DC	*Eucalyptus* spp.
6132	2014	Nariño	*Pinus* spp.

* Strain was not viable for culture; CSF: Cerebrospinal fluid; BAL: Bronchoalveolar lavage.
